# Differential Transcription and Alternative Splicing in Cotton Underly Specialized Defense Responses Against Pests

**DOI:** 10.3389/fpls.2020.573131

**Published:** 2020-09-15

**Authors:** Dian-Yang Chen, Qiu-Yi Chen, Dan-Dan Wang, Yu-Pei Mu, Mu-Yang Wang, Ji-Rong Huang, Ying-Bo Mao

**Affiliations:** ^1^ Shanghai Key Laboratory of Plant Molecular Sciences, College of Life Sciences, Shanghai Normal University, Shanghai, China; ^2^ CAS Key Laboratory of Insect Developmental and Evolutionary Biology, CAS Center for Excellence in Molecular Plant Sciences, Shanghai Institute of Plant Physiology and Ecology, University of CAS, Chinese Academy of Sciences, Shanghai, China; ^3^ School of Life Science and Technology, ShanghaiTech University, Shanghai, China

**Keywords:** plant defense, *Apolygus lucorum*, *Helicoverpa armigera*, jasmonate signaling, alternative splicing

## Abstract

The green mirid bug (*Apolygus lucorum*) and the cotton bollworm (*Helicoverpa armigera*) are both preferred to live on cotton but cause different symptoms, suggesting specialized responses of cotton to the two insects. In this study, we investigated differential molecular mechanisms underlying cotton plant defenses against *A. lucorum* and *H. armigera via* transcriptomic analyses. At the transcription level, jasmonate (JA) signaling was dominated in defense against *H. armigera* whereas salicylic acid (SA) signaling was more significant in defense against *A. lucorum*. A set of pathogenesis-related (PR) genes and protease inhibitor genes were differentially induced by the two insects. Insect infestations also had an impact on alternative splicing (AS), which was altered more significantly by the *H. armigera* than *A. lucorum*. Interestingly, most differential AS (DAS) genes had no obvious change at the transcription level. GO analysis revealed that biological process termed “RNA splicing” and “cellular response to abiotic stimulus” were enriched only in DAS genes from the *H. armigera* infested samples. Furthermore, insect infestations induced the retained intron of GhJAZs transcripts, which produced a truncated protein lacking the intact Jas motif. Taken together, our data demonstrate that the specialized cotton response to different insects is regulated by gene transcription and AS as well.

## Introduction

Plants are sessile organisms and encounter a wide variety of herbivores during their life cycle. Insects have different mouthparts and feeding habits and they also secrete different active molecules to plants (Hogenhout and Bos, 2011; Chen et al., 2019; Jiang et al., 2019; Xu et al., 2019). Plants can distinguish the different insect infestations and make acute and specialized responses for survival. A set of protease inhibitor genes can be quickly activated in plants by the leaf-chewing Lepidopterans (Haq et al., 2004; Bezzi et al., 2010; Kuwar et al., 2015). The phloem-feeding insects, like whiteflies and aphids, induce the expressions of pathogenesis-related (PR) genes that are associated with disease resistance in plants (De Vos et al., 2006). Jasmonate (JA) and salicylic acid (SA) are two important defense hormones that coordinate with multiple signaling to form complex regulatory networks (Howe et al., 2018; Erb and Reymond, 2019). It is reported that JA is involved in plant defense against leaf-chewing insects, mesophyll feeder, and necrotrophic pathogens (Howe and Jander, 2008; Furstenberg-Hagg et al., 2013; Machado et al., 2016). SA is generally related to plant defense against sap-sucking insects and biotrophic pathogens (Pieterse et al., 2012). In many cases, JA- and SA-mediated signaling pathways are not separated but integrated. The differential responses of phytohormones in plants caused by insect infestations lead to highly specialized responses at the transcriptional level. Although JA/SA is well known in plant defense, the comparison of the detailed differential reactions of JA/SA signaling caused by insects with different feeding guilds is limited. Alternative splicing (AS) exists ubiquitously in eukaryotes, including animals, plants and fungi. There are four main types of AS in plants: skipping exon (SE), retained intron (RI), alternative 5’ splice site (A5SS) and alternative 3’ splice site (A3SS) (Dong et al., 2018a; Breitbart et al., 1987). One gene can produce multiple different mRNA transcripts and causes variant protein products through AS regulation. In plants, AS are found related to plant growth, development, and light morphology (Staiger and Brown, 2013; Hartmann et al., 2016). It also links to stress responses (Laloum et al., 2018; Calixto et al., 2018; de Francisco Amorim et al., 2018) and maintenance of the mineral nutrient homeostasis (Dong et al., 2018). JAZ proteins are the main repressors of the JA signaling (Chini et al., 2007; Sun et al., 2017; Howe et al., 2018). Multiple JAZ coding genes with variant splicing transcripts in Arabidopsis implies that AS regulation is involved in JA-mediated defense against insects (Zhang et al., 2017; Wu et al., 2019). It has been reported that AS patterns differ in two maize lines when responsed to aphid (Song et al., 2017). However, little is known about the global dynamics of AS and its function in plant response to different insects. Cotton is a global important fiber crop. The green mirid bug (*Apolygus lucorum*) and the cotton bollworm (*Helicoverpa armigera*) are two main pests with different mouthparts and feeding habits in cotton fields. The *H. armigera* larva belongs to leaf-chewing insects while *A. lucorum* is a mesophyll feeder that punctures into a leaf and consumes mesophyll cells. The cotton plant symptoms caused by the two insects were quite different. The *H. armigera* larvae infestation caused wounding damages and a large amount of leaf tissue losses. On the other hand, the *A. lucorum* affected cotton plants exhibited unique symptoms including leaf wilting, necrotic plaques and abnormal leaf development (**Supplementary Figure S1**). In this study, we investigate the different defense responses to *H. armigera* and *A. lucorum* in cotton *via* transcriptomics analysis. Our data reveal extensive differences between cotton responses to two different insects at transcriptome levels, including gene expression and AS patterns. These data provide new insight into the regulatory elements on plant-insect interactions.

## Materials and Methods

### Plant and Insect Cultures

Cotton (*Gossypium hirsutum* cv. R15) plants were grown in a climate chamber at 28°C, on a 16-h light/8-h dark photoperiod. *H. armigera* was reared in the laboratory at 25°C and 70% relative humidity with 14-h light/10-h dark photoperiod on artificial diet (Sinica et al., 2010). *A. lucorum* were reared on kidney beans in the laboratory at 22°C and 70% relative humidity with 12-h light/12-h dark photoperiod. For insect feeding treatment on the cotton leaf, the cotyledons of cotton seedlings that grow up to around 15 days were covered with plastic bags and each plastic bag contained 2 third-instar *H. armigera* larvae (HA) or 5 adult *A. lucorum* (AL). The cotyledons covered with empty plastic bags were used as control (CK). After 24 h of treatment, the cotyledon samples of HA, AL, and CK were collected for RNA extraction.

### Hormone Treatment

SA (Sigma, USA) and methyl jasmonic acid (MeJA) (Sigma, USA) were dissolved in ethanol and configured as 1M and 0.25M storage solutions, respectively. Cotton cotyledons of 15 days seedlings were sprayed with SA (1 mM) and MeJA (250 μM) solution and collected at 1, 4 h, and 24 h post spray. Two independent tests with four biological replicates were performed.

### Cotton Sample Preparation and RNA Sequencing

For transcriptome sequencing and gene expression analysis, each cotyledon samples of HA, AL, and CK have three biological replicates. Every replicate contained 6 cotton cotyledons. Total RNAs were isolated with CTAB (cetyltrimethylammonium bromide) extraction solution (2% CTAB, 0.1 M Tris, 20 mM EDTA, 1.4M NaCl, pH = 9.5) (Stewart and Via, 1993), precipitated by 2 M LiCl (Yang et al., 2010). DNase I was used to removing genomic DNA. RNA concentrations were determined by Nanodrop 2000 (NanoDrop products, USA) and RNA integrity was checked by Agilent 2100 (Agilent Technologies, USA). About 10 μg of the total RNA from each sample, was used to enrich poly(A) mRNA using oligo-dT magnetic beads (Invitrogen, USA), followed by fragmentation into 100–400 nt sizes, which were used to synthesize cDNAs with random hexamer primers (Invitrogen, USA). Agilent 2100 Bioanalyzer and ABI StepOnePlus Real-Time PCR System were used to quantify and qualify all libraries. Then, paired-end RNA-seq libraries were prepared following the Illumina’s library construction protocol. The libraries were sequenced on Illumina HiSeq 4000 platform (Illumina, USA) at 1Gene (Hangzhou, China).

### Sequence Alignment and Differential Expression Transcript Identification

Before mapping, 2 × 200 bp paired-end raw reads from each cDNA library were processed to remove low-quality sequences (Q < 20, reads of N > 5% and adaptors). SOAPaligner/SOAP2 (Li R. et al., 2009) (-m 0 -x 1,000 -s 40 -l 32 -v 5) was used to align the clean reads from each library to the reference cotton transcripts (Tianzhen et al., 2015). The RSEM (Li and Dewey, 2011) package was applied to calculate the normalized gene expression values of FPKM (fragments per kilobase per million mapped reads). A total of 23,729 well-expressed genes (unique reads > 20, coverage rate > 80%, FPKM > 5 in all the three replicates of at least one sample group) were screened for downstream analysis. Differential expressed genes were analysed by DEseq2 (Love et al., 2014) (padj < 0.001, lfcSE < 0.5).

TBtools (Chen et al., 2020) was used for GO enrichment analysis based on hypergeometric test, and Benjamini-Hochberg procedure was used for multiple testing. Set the cotton GO annotation (Ashburner et al., 2000; The Gene Ontology Consortium, 2018) according to the closest Arabidopsis gene (Tianzhen et al., 2015) for each cotton gene as a GO background.

For cluster analysis, amino acid sequences were aligned using ClustalX (Thompson et al., 1997), and the phylogenetic trees were constructed using the maximum parsimony method MEGA 5.1 (Tamura et al., 2011) with default settings and 1,000 bootstrap replicates.

### Quantitative RT-PCR (qRT-PCR)

Total RNAs (1 μg) were used for cDNA synthesis by Genomic DNA Removal and cDNA Synthesis kit (Transgene, China) and the cDNA products were diluted 10 times. The qRT-PCR was performed on a Bio-Rad CFX Connect Real-Time PCR system (Bio-Rad, USA) using the SYBR Green PCR Mix (Bio tool, USA), according to the manufacturer’s instructions for standard two-step amplification program. Multiple biological replicates (n > 3 times; see in relevant figure legends) with technical duplicates were performed. The relative expression of genes was calculated using the 2^−ΔΔ^Ct method. Cotton HIS3 (Tian et al., 2018) was used as the internal standard. The oligonucleotide primers used in this investigation are given (**Supplementary Table S1**).

### Analysis the Effects of Protease Inhibitors on Larval Growth and Protease Activity

The ORF of Gh_Sca005135G01(5135) and Gh_A11G1177(1177), in frame, were fused to the maltose-binding protein (MBP) tag of the expression vector pMAL-C5X (New England Biolabs), and then transformed into *E. coli* BL21 strain for prokaryotic expression. The oligonucleotide primers used in this investigation are given (**Supplementary Table S1**). The recombinant proteins were induced by 0.2 mM IPTG at 22°C for 16 h and affinity-purified following the manufacturer manual.

For analysis of the inhibition effects on midgut protease activity by protease inhibitor (Mao et al., 2013), 1 μl *H. armigera* midgut fluid was mixed with 9 μl of 50 mM Tris-HCl (pH 8.0) and incubated with10 μl purified recombinant proteins 5135, 1177, and MBP (2 mg/ml), respectively, at 28°C. After 30-min incubation, 40 μl 1% azocasein was added and incubated for another hour at 28°C. Then, 40 μl of 10% trichloroacetic acid was added to stop the reaction and incubated at room temperature for 1 h. The mixture was then centrifuged at 16,000 g for 10 min to remove the undigested azocasein. The supernatant was collected and mixed with equal volume 1 M NaOH and then the optical density was determined at 450 nm by Nanodrop 2000 (NanoDrop products, USA). Each protease activity assay contains 6–8 biological replicates.

For analysis the 5135 effects on the *H. armigera* growth, about 500-ml culture solution (OD = 1.0) of *E. coli* cells expressing 5135 and MBP, respectively, were centrifuged and the precipitate was mixed with 50 g artificial diet with indicated nutrition. For the artificial diet with 1/2 nutrition, the amount of wheat germ and casein in the artificial diet was reduced to half. The 2nd instar larvae of *H. armigera*, which were in a similar growth stage, were selected for experiments. After 5 days of feeding, the larvae weight was recorded, and the midgut fluid was extracted for protease activity assay. The insect feeding tests were repeated for three times (independent experiments).

### AS Analysis

The clean data of CK, HA, and AL were subject to cufflinks (Trapnell et al., 2010) using the original GTF file (Tianzhen et al., 2015) as a reference to get the total possible mRNA variants. Then, the mRNA variants detected in all the three replicates of at least one sample group were selected and merged with the original GTF file (Tianzhen et al., 2015) to create a high quantity GTF file. All the clean data were mapped to the cotton genome (Tianzhen et al., 2015) by HISAT2 v2.1.0 (Kim et al., 2015) using the high quantity GTF file as a reference and followed by rMATS analysis to obtain the probable AS events. Well-expressed AS genes were screened by the IJC (inclusion junction counts) and SJCs (skipping junction counts) values under the rules (**Supplementary Table S2**). We compared the AS events in CK with that in HA and AL, respectively. The differential AS (DAS) events were calculated with the threshold of |Δ Percent spliced in (PSI) | > 0.05, FDR < 0.05 and standard deviation of PSI < 0.01.

### Data Accessibility

All the raw sequence data of this article are deposited in the NCBI (BioProject accession number: PRJNA600707).

## Results

### Overall Impacts of *Helicoverpa armigera* and *Apolygus lucorum* Infestations on Cotton

**Figure 1:**
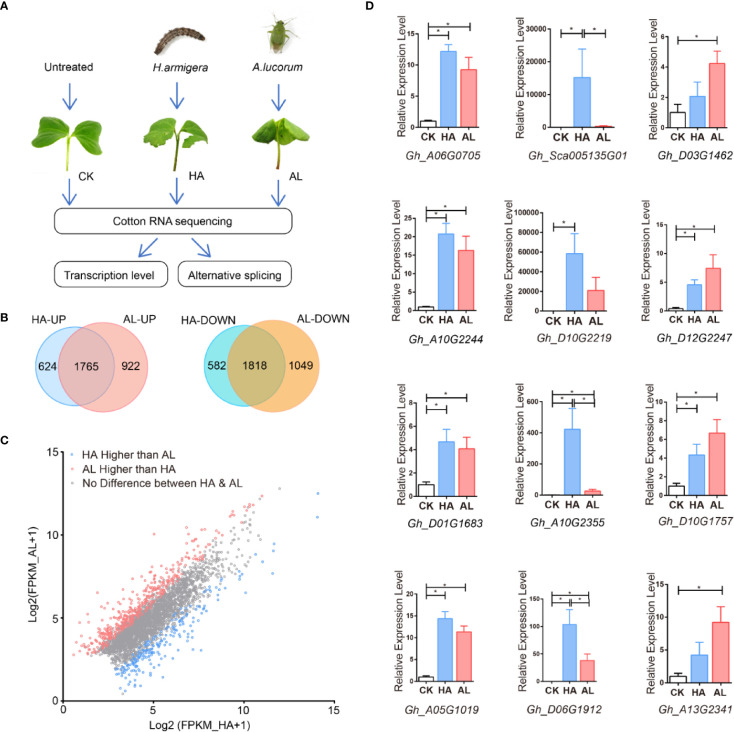
RNA sequencing (RNAseq) using sample groups from the untreated, *H. armigera* and *A. lucorum* infested cotyledons. **(A)** The flow diagram of the sample collection, sequencing, and analysis. The cotyledons were treated with *H. armigera* (HA) and *A. lucorum* (AL) for one day and the untreated cotyledons were used as control (CK). Total RNA of CK, HA, and AL samples with biological triplicates were sequenced and analyzed at both transcription and splicing levels. **(B)** The Venn diagrams of up- and down-regulated genes in cotton by *H. armigera* and *A. lucorum*. The overlapped regions stand for genes up- or down-regulated by both insects. **(C)** Scatter plot analysis of the total up-regulated genes by either insect feedings. The X- and Y-axis stand for the gene expression [Log2(FPKM+1)] in HA and AL, respectively. The blue and red spots indicate the genes with higher induction levels in HA and AL respectively (padj < 0.001). The gray spots indicate that the induction of these genes has no difference between HA and AL. **(D)** RT-PCR analysis of the 12 up-regulated DEGs from the RNA-seq results. Every four genes of both highly induced (first column), more highly induced in HA (second column) and more highly induced in AL (third column) were selected. Cotton cotyledons were treated as described in A. *GhHIS3* was used as the internal standard. The expression in CK was set to 1. Error bar means ± SEM (n = 5 biological replicates). The results were consistent with the RNA-seq results. *T*-test, *P < 0.05.

To gain deeper insights into cotton plant defenses against *H. armigera* and *A.lucorum*, we performed RNA sequencing (RNAseq) using sample groups from the untreated (control, CK), *H. armigera* (HA), and *A. lucorum* (AL) infested cotyledons (**Figure 1A** and **Supplementary Table S3**). The global gene expression profiles had high uniformity within the 3 biological replicates of the same treatment and were quite different among the CK, HA, and AL (**Supplementary Figures S2A, B**). Compared to the control, there were 4,789 differentially expressed genes (DEGs) (2,389 up- and 2,400 down-regulated) in HA and 5,554 DEGs (2,687 up- and 2,867 down-regulated) in AL (**Supplementary Figure S2C**). The DEGs caused by the two insects were highly shared. A total of 1,765 genes were induced and 1,818 genes were reduced by both insects (**Figure 1B**). The down-regulated genes both in HA and AL are enriched in GO items such as “response to light stimulus” and “tetrapyrrole biosynthetic process”, showing the common effects on plant photosynthetic function and secondary metabolism by the two insects. The genes only down-regulated in HA was enriched in “regulation of chlorophyll metabolic process” and the genes only down-regulated in AL was enriched in “microtubule-based process” and “plant-type primary cell wall biogenesis” showing the differential down-regulation of gene expression in cotton response to the two insects (**Supplementary Figure S2D** and **Table S4**). For insect up-regulated DEGs, the following terms “response to wounding”, “response to chitin”, “salicylic acid biosynthetic process”, and “jasmonic acid-mediated signaling pathway” are enriched both in up-regulated DEGs of HA and AL (**Supplementary Table S5**). Although a large proportion of DEGs was the same between HA and AL, from the total DEGs up-regulated by either *H. armigera* or *A.lucorum*, scatter plot analysis displayed that 395 showed significant higher induction in AL whereas the inductions of 205 DEGs were significantly higher in HA (**Figure 1C**). Twelve up-regulated DEGs from the RNA-seq analysis were selected for further confirmation by qPCR analysis. As expected, we got consistent results with that obtained from the RNA-seq (**Figure 1D**).

### JA and SA Had Different Contributions in the Defense Against *H. armigera* and *A. lucorum*

**Figure 2:**
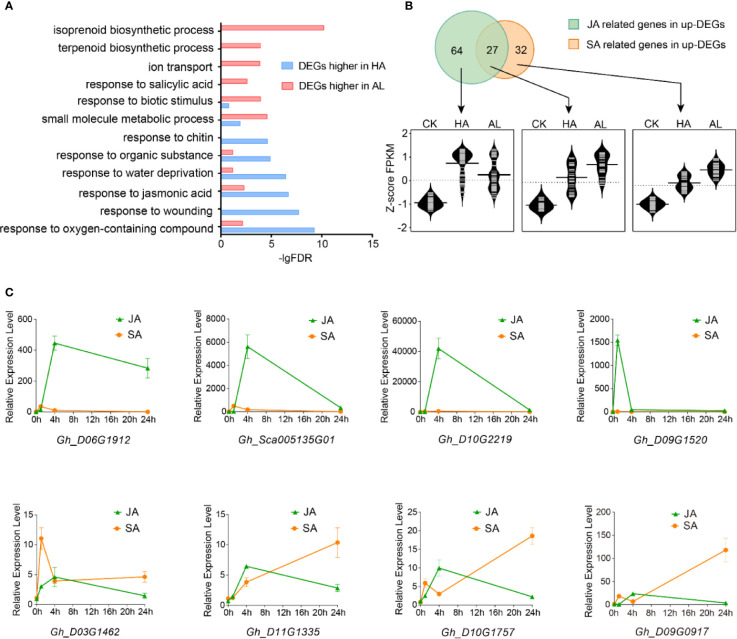
Different contributions of JA and SA in cotton defense against insects. **(A)** GO enrichment of the DEGs with higher inductions by *H. armigera* (HA, blue) and by *A. lucorum* (AL, red) were analyzed, respectively. **(B)** RNA-Seq analysis of JA and SA related gene expressions. The up part is the Venn diagrams of the induced DEGs related to JA and SA. The green part stands for the genes related to JA, the orange part stands for the genes related to SA and the overlapped part stands for the genes both related to JA and SA. The bottom part is the violin plot of JA and SA related genes in CK, HA, and AL.The FPKM of each gene was normalized by z-score. The average z-scoe of each gene was showed in the violin plot and the long horizontal line represented for the median. The detailed JA and SA related gene information were listed in **Supplementary Table S7**. **(C)** qRT-PCR analysis of the selected DEGs which were more highly induced by *H. armigera* (up) and by *A. lucorum* (bottom) from RNA-Seq data. The cotton cotyledons were treated with JA and SA respectively and collected at 1, 4, and 24 h post treatment. The expression of indicated genes was detected by qRT-PCR. *GhHIS3* was used as the internal standard. The expression of untreated cotton leaves (0h) was set to 1. Error bar means ± SEM (n = 4 biological replicates).

To further investigate the different responses in cotton against the two insects, the DEGs with significant higher inductions by *H. armigera* and by *A. lucorum* were subject to GO assay separately. Interestingly, the items including “response to oxygen-containing compound”, “response to wounding”, “response to jasmonic acid”, and “response to chitin” were enriched (p < 0.001) only in the DEGs with higher inductions by *H. armigera* while the terms: “terpenoid biosynthetic process”, “response to salicylic acid” were highly enriched only in DEGs with higher inductions by *A. lucorum* (**Figure 2A** and **Supplementary Table S6**).

From the total induced DEGs by either *H. armigera* or *A.lucorum*, 91 genes are related to the jasmonic acid pathway and 59 genes are related to the SA pathway. Venn diagram analysis showed that 64 genes were distributed only in JA-related items (JA-only), 27 genes were distributed in both JA- and SA-related items (JA/SA), and 32 genes were distributed only in SA-related items (SA-only). These JA-only DEGs showed higher induction in HA than in AL whereas JA/SA and SA-only DEGs were more highly induced in AL than in HA (**Figure 2B** and **Supplementary Table S7**). We selected 8 DEGs which were both induced by the two insects to analyze the impacts of JA and SA signaling on their expressions. The four selected DEGs with higher induction by *H. armigera* could be strongly induced by MeJA treatment but not by SA treatment, whereas the rest four DEGs with higher induction by *A. lucorum* could be induced either by MeJA or SA treatment and the inductions were more dominant in SA than in MeJA treatment (**Figure 2C**). These data supported that JA and SA were differentially contributed to plant defense. JA was a dominant regulator in defense against *H. armigera* whereas SA might be more important in defense against *A.lucorum*.

### More Significance Induction of PR Genes by *A. lucorum*

**Figure 3:**
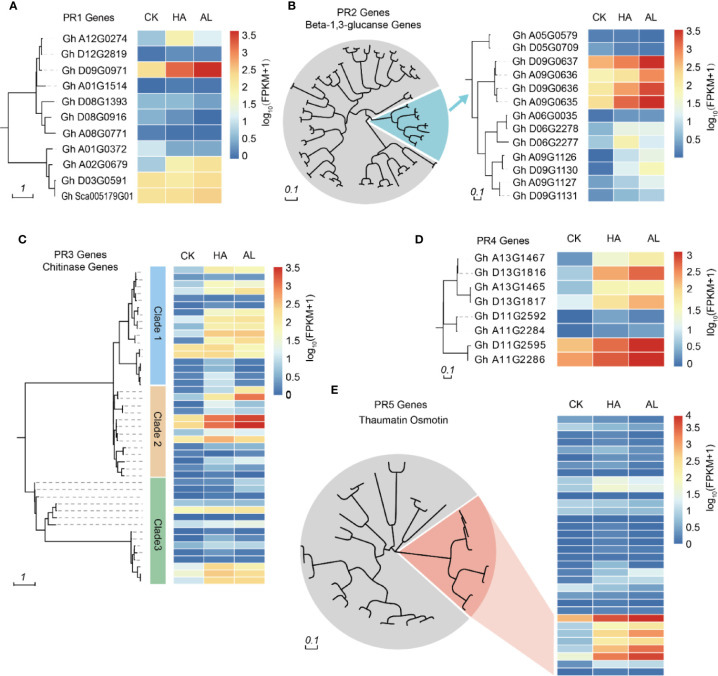
Phylogenetic analysis of PR genes and their transcription levels. Cluster analysis of PR1 **(A)**, PR2 **(B)**, PR3 **(C)**, PR4 **(D)**, and PR5 **(E)** family members in cotton. The heatmap in the right indicated the expression levels of the corresponding PR genes in untreated (CK), *H.armigera* (HA) and *A. lucorum* (AL) infested samples from the RNA-seq results. The detailed gene information was listed in **Supplementary Table S8**.

Plant PR proteins are involved in various types of pathogen infections such as viruses, bacteria, and fungi (Lamb et al., 1989; Ali et al., 2018). Some PR proteins had also been reported to have insecticide activity (Singh et al., 2018). Based on their amino acid sequence similarity, enzymatic activity, or other biological properties (van Loon et al., 2006; Breen et al., 2017), PR proteins are classified into 17 groups of which the PR1, PR2, PR3, PR4, and PR5 are the five groups discovered firstly (Kitajima and Sato, 1999). PR1 protein was discovered in tobacco in response to tobacco mosaic virus (TMV) infection (Cornelissen et al., 1986) and its homologs have been identified in barley, tomato, corn, and rice (Niderman et al., 1995; Liu and Xue, 2006). The PR1 family contains the most abundant PR proteins which are induced by pathogen infections (Breen et al., 2017). We found 11 PR1 homolog genes in cotton and two of them can be induced by insect infestations (**Figure 3A**). Plant β-1,3-glucanases belong to the PR-2 family and reportedly play an important role in plant defense responses (Balasubramanian et al., 2012; Xie et al., 2015) and other biological processes such as pollen development (Wan et al., 2011), seed germination (Leubner-Metzger and Meins, 2000), and cold response (Hincha et al., 1997). These highly regulated enzymes catalyze the hydrolytic cleavage of β-1,3-glucans abundantly present in plant cell walls (Hoj and Fincher, 1995). Among the 82 cotton β-1,3-glucanase genes, seven genes in one branch were significantly induced by the insect feedings (**Figure 3B**). Chitin is a main component of the fungal cell wall and exoskeleton elements of insects. The PR3 family belongs to chitinase. In cotton, 45 chitinases were found and 16 of them in different clusters were induced by insects (**Figure 3C**). The PR4 family can be divided into two classes according to the functional domain: Class 1 is endochitinases because they can bind to chitin and exhibit chitinase activity and class 2 has RNase activity (Ponstein et al., 1994; Brunner et al., 1998; Li X. et al., 2009). There were five of the eight PR4 proteins in cotton which were significantly induced by insect feeding (**Figure 3D**). The PR5 protein family has high amino acid homology to sweet-tasting protein/thaumatin, including thaumatin-like protein and osmotin (Sinha et al., 2014). The expression levels of thaumatin-like genes in cotton did not change much by insect infestation, while 6 osmotin like genes were significantly induced (**Figure 3E**). From the insect-induced PR genes, most of them were induced by both *H. amigera* and *A. lucorum* indicated that these PRs might involve in cotton defense against both insects. Notably, the inductions of 10 PR genes were obviously higher in AL than in HA (**Supplementary Figure S3**) while only one showed higher induction in HA, suggesting more significant roles of PRs in the defense against *A. lucorum* than *H. amigera* (**Supplementary Table S8**).

### The Two Insects Caused Differential Inductions of Protease Inhibitor Genes

**Figure 4:**
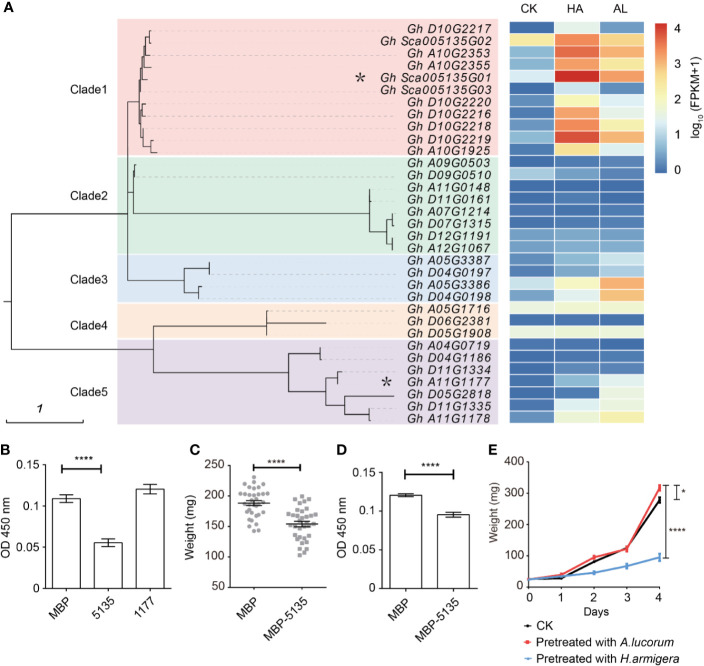
Differentially expressed protein inhibitors by *H. armigera* and *A. lucorum*. **(A)** Cluster analysis revealed that the serine protease inhibitors (SPIs) in cotton can be divided into five clades. The heatmap in the right indicated the expression level [lg(FPKM + 1)] of the corresponding SPI genes in untreated (CK), *H.armigera* (HA) and *A. lucorum* infested (AL) samples from the RNA-seq results. The SPI genes with an asterisk were used for the functional analysis. **(B)** The total proteinase activities of the larval gut fluids were inhibited by Gh_Sca005135G01(5135) instead of Gh_A11G1177 (1177). Gut fluids were incubated with the prokaryotically expressing proteins of MBP, 5135, and 1177, respectively. After incubation for 30 min, the proteinase activities of the gut fluids were detected. Error bar means ± SEM (n = 6 biological replicates). *T*-test, ****P < 0.0001. **(C)** Oral ingestion of 5,135 inhibited larval growth. The 2^nd^ instar larvae were fed with the artificial diet (1/2 nutrition) mixed with the *E. coli* cells expressing MBP, 5,135 respectively for 5 days and the larval weight was recorded. Error bar means ± SEM (n = 30–35). *T*-test, ****P < 0.0001. **(D)** The total proteinase activities of the gut fluids from the larvae were determined. Error bar means ± SEM (n = 8 biological replicates). *T*-test, ****P < 0.0001. **(E)** The *H. armigera* larvae pre-fed plants showed enhanced resistance to its second feeding. The 15 days old cotton seedlings were pre-fed with *H. armigera* and *A. lucorum* for one day. The 2^nd^ instar larvae were fed with the untreated (CK) and the insect pre-fed cotton cotyledons for 4 days and the weight of each individual was recorded. Error bar means ± SEM (n = 20–24). The weight of larvae on 4^th^ day is analyzed by *T*-test. *P < 0.05, ****P < 0.0001. The insect feeding tests **(C, F)** were repeated for three times (independent experiments) and the results were consistent.

In plants, protease inhibitors are involved in many physiological processes, including promoting storage proteins, inhibiting endogenous enzyme activity, regulating apoptosis, programmed cell death, and insect resistance (Haq et al., 2004; Grosse-Holz and van der Hoorn, 2016). Protease inhibitors can be divided into four major families: cysteine protease inhibitors, non-metalloproteinase inhibitors, aspartic protease inhibitors, and serine protease inhibitors (SPIs) (Laskowski and Kato, 1980), of which the SPIs are the most extensively studied (Valueva and Mosolov, 2004; Rawlings et al., 2018). Some SPIs have been identified to have insecticide activities and applied for developing insect-resistant transgenic plants (Jongsma et al., 1995; Cloutier et al., 2000; Clemente et al., 2019). Cotton is one of the most preferred host plants of cotton bollworm and has a large number of SPIs; however, none of the SPIs has been identified involving in insect resistance. There were 33 SPI genes which were existed in our RNA-seq database and were classified into five clades (**Figure 4A**). Among them, most SPI genes of the clade 1, clade 3, and part of the clade 5 could be induced by both insect feeding. Interestingly, induced SPI genes of clade 1 exhibited much higher induction by *H. armigera* whereas induced SPI genes of clade 3 and clade 4 were more highly induced by *A. lucorum* (**Figure 4A**). This indicated that there was a clear association between the phylogenetic evolutions of protease inhibitors and their induction patterns by different insects. *Gh_Sca005135G01* from the clade 1 was more highly induced and its transcripts were the most abundant among SPIs in cotton by *H. amigera* and *Gh_A11G1177* in clade 5 was more highly induced by *A.lucorum*. These two SPIs were fused with MBP and expressed in *Escherichia. coli*. After incubation with prokaryotically expressed 5135 protein, the protease activity of the cotton bollworm midgut fluid was reduced by about 30%. However, when Gh_Sca005135G01 was instead of Gh_A11G1177 for assay, there was little effect on the protease activity of midgut fluid (**Figure 4B**). When the 2nd instar larvae of *H. armigera* were fed with the standard artificial diet mixed with *E. coli* cells expressing MBP and Gh_Sca005135G01, respectively, the larvae growth rates were similar (**Supplementary Figure S4**). Larvae fed with the standard artificial diet are growing more faster than that fed with cotton leaves. In view of that the adverse effects of protein inhibitiors might be reduced due to the adequate nutrition in artificial diet, we reduced the nutrition of the artificial diet to half for testing. Under such condition, the weight of larvae fed with the *E. coli* cells expressing Gh_Sca005135G01 was reduced by ~25% compared with that fed with MBP-expressed *E. coli* cells (**Figure 4C**). Accordingly, the protease activity of the midgut fluid extracted from the larvae which were fed with the Gh_Sca005135G01 expressing *E. coli* cells was decreased (**Figure 4D**). Combined with the result that the *H. armigera* larvae pre-fed plants showed predominantly enhanced resistance to its second feeding, while *A. lucorum* nymphs pre-feeding only have a little adverse effects on *H. armigera* (**Figure 4E**), we inferred that Gh_Sca005135G01 was toxic to cotton bollworm by blocking protease activities in digestion process on the condition when the larvae were raised on the diet with low nutrition.

### The Impacts of Insect Feedings on Gene AS Profile

**Figure 5:**
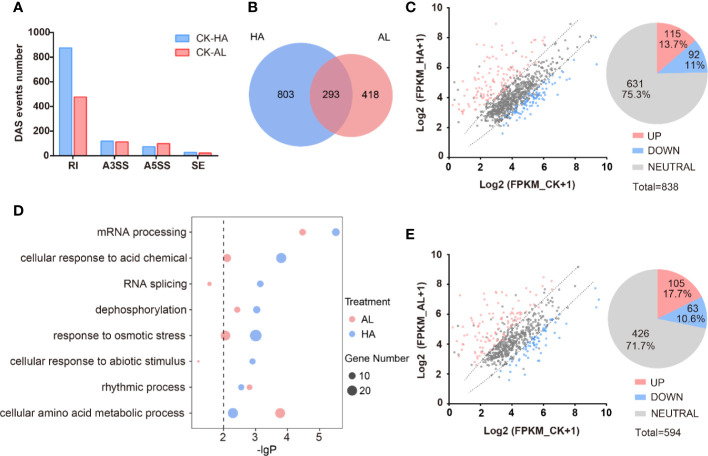
The alternative splicing profile of cotton was affected by the two insect infestations. **(A)** The differential AS (DAS) events caused by the *H. armigera* and *A. lucorum* infestations in cotton. **(B)** The Venn diagrams of DAS events caused by the *H. armigera* (HA, blue) and *A. lucorum* (AL, red) infestations. The overlapped region stands for the DAS events caused by both insects. **(C, E)** Scatter plot analysis of the transcription levels of the DAS genes in HA **(C)** and in AL **(E)**. The X-axis stands for the gene expression (Log2(RPKM+1)) in CK and the Y-axis stands for the gene expression (Log2(RPKM+1)) in HA **(C)** and in AL **(E)**. **(D)** GO enrichment analysis of the DAS genes from HA samples (blue spots) and from AL samples (red spots) were analyzed respectively. The size of the spot represents the number of DAS genes.

We identified a total of 11,023 AS events from the RNA-seq data of CK, HA, and AL sample groups. Among them, RI events (7,676) occupied the maximum proportion (~70%). A3SS events (1,844) and A5SS events (1,216) account for ~17% and ~11%, respectively. The proportion of SE events was extremely low, accounting for less than 3% of the total (**Supplementary Figure S5**). The distribution feature of the different types of AS events were similar to that of the Arabidopsis, rice and maize (Marquez et al., 2012; Mei et al., 2017; Dong et al., 2018). There are 1,514 DAS events in total caused by either *H. armigera* or *A. lucorum* infestations and RI was the dominant type as expected (**Figure 5A** and **Supplementary Table S9**). The numbers of differential RI events in HA were almost twice that in AL while the amounts of differential A3SS, A5SS, and SE events were similar between HA and AL. The DAS events caused by *H. armigera* and *A. lucorum* were largely different and only 293 DAS events were shared (**Figure 5B**). The transcriptional levels of the DAS genes were largely unaffected by either of insect infestations. For DAS genes in HA, only 115 were up-regulated and 92 were down-regulated while 631 had no obvious change in the transcription level by *H. armigera* (**Figure 5C**). Similarly, the transcription levels of most DAS genes (426) in the AL group were not affected either, only 105 up- and 63 down-regulated by *A. lucorum* (**Figure 5E**). These results suggest that insect infestation regulated a set of plant defense genes at the splicing level but not at the transcription level.

GO enrichment analysis of the DAS genes of HA and AL revealed that the “rhythmic process” items had similar enrichment levels in HA and AL; the “mRNA processing”, “cellular response to acid chemical”, “dephosphorylation”, and “response to osmotic stress” items are both enriched in HA and AL. However, more significance was observed in HA than in AL (**Figure 5D** and **Supplementary Table S10**). The “RNA splicing” and “cellular response to abiotic stimulus” items are found only enriched in HA while the “cellular amino acid metabolic process” is the only item that had a higher enrichment level in AL (**Figure 5D**). Notably, all the above items were not enriched in the DEGs, suggesting different roles of transcription regulation and AS regulation in cotton defense against insects.

### Functional Significance of AS in Cotton Defense Against Insects

**Figure 6:**
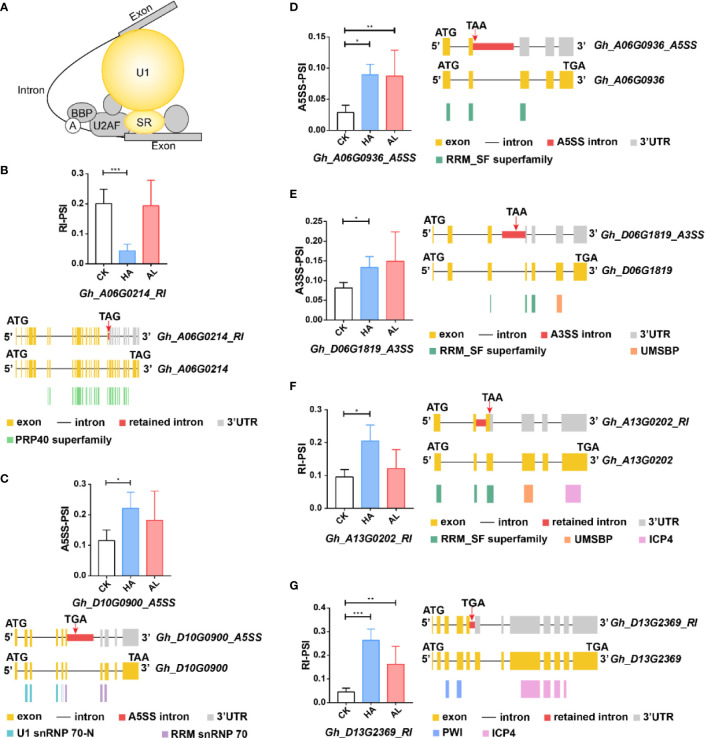
The differential alternative splicing of the splicing-related genes caused by the insect infestations. **(A)** The DAS genes involved in the splicing pathway were mainly U1-related and SR proteins which were colored in yellow. **(B**–**G)** The expression level of the representative variant splicing form in cotton by *H. armigera* (HA) and *A. lucorum* (AL) infestations. Error bar means ± SEM (n = 3 biolgical replicates). *FDR < 0.05; **FDR < 0.01; ***FDR < 0.001. The conserved domains of the DAS genes were marked in the corresponding color. ATG and TGA/TAA stand for initiation and termination codon respectively. The differential alternative splicing regions were marked in red. Yellow and gray boxes stand for exons and 3’UTR, respectively. The black line represents intron.

The DAS genes from HA enriched in the splicing items were mainly U1 snRNP and SR-related genes (**Figure 6A**). *Gh_A06G0214* encoded a U1 snRNP-related protein and its RI transcript (*Gh_A06G0214-RI*) resulted in a truncated protein with the partial PRP40 domain (**Figure 6B** and **Supplementary Table S11**) which was a splicing factor domain involved in RNA processing and modification (Ester and Uetz, 2008). *Gh_D10G0900* gene encoded the U1 snRNP 70K subunit, and the protein product of its A5SS transcript (*Gh_D10G0900-A5SS*) lacked the RNA recognition domain (RRM snRNP 70) (**Figure 6C**). The expressions of *Gh_A06G0214-RI* and *Gh_D10G0900-A5SS* were largely affected by *H. armigera*, whereas no significant change was observed by *A. lucorum* (**Figures 6B, C**). For the SR related protein-coding genes, *Gh_A06G0936* belongs to the SC35 subfamily and *Gh_D06G1819* and *Gh_A13G0202* belong to Two-Zn-knuckles–type subfamily SR proteins (Iida and Go, 2006). The *H. armigera* induced the AS transcripts (*Gh_A06G0936_A5SS*, *Gh_D06G1819_A3SS* and *Gh_A13G0202_RI*) of which the protein products lacked the integral RRM_SF superfamily domain (**Figures 6D–F**). *Gh_D13G2369* is a splicing factor PWI domain-containing protein. The RI transcript (*Gh_D13G2369-RI*) which encoded a protein lacking the ICP4 superfamily domain was significantly induced in HA (**Figure 6G**). Although some DAS of the above SR genes were also observed in AL, the significance was less than that in HA (**Figures 6D–G**).

**Figure 7:**
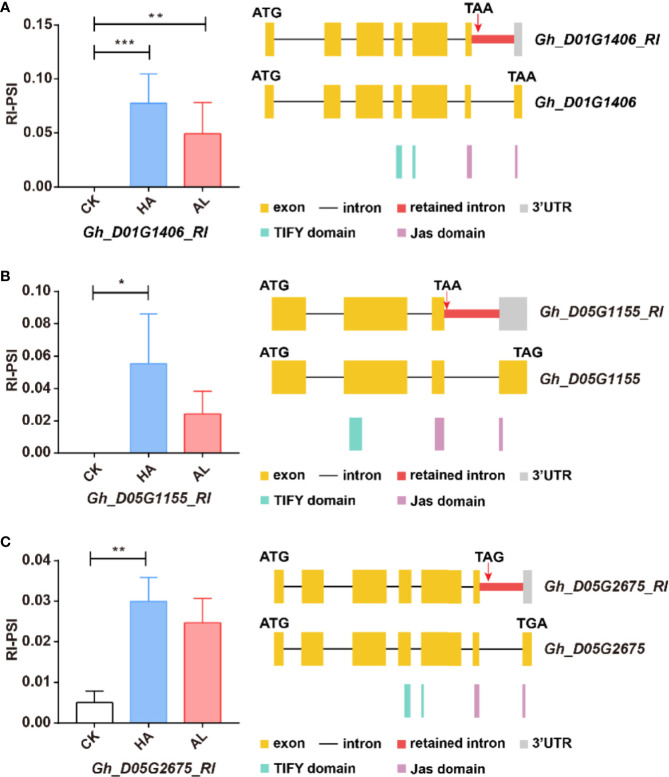
The RI variants of the GhJAZ genes were induced by insect infestations. *Gh_D01G1406* **(A)**, *Gh_D05G1155* **(B)**, and *Gh_D05G2675* **(C)** encoded GhJAZ proteins, and the RI variants were promoted by *H. armigera* (HA) and *A. lucorum* (AL) infestations compared with the untreated cotton leaves (CK). Error bar means ± SEM (n = 3 biolgical replicates). *FDR < 0.05; **FDR < 0.01; ***FDR < 0.001. The Jas motif was marked in purple. ATG and TGA/TAA stand for initiation and termination codon respectively. The differential alternative splicing regions were marked in red. Yellow and gray boxes stand for exons and 3’UTR, respectively. The black line represents intron.

The JA ZIM-domain proteins are the main repressors of JA signaling (Chini et al., 2007; Sun et al., 2017). AS of multiple JAZ genes is observed in Arabidopsis (Chung and Howe, 2009; Chung et al., 2010; Moreno et al., 2013). For example, AtJAZ10.1 has a complete jas domain, while the jas motif of AtJAZ10.3 and AtJAZ10.4 is partially destroyed or missing, making them insensitive to JA-Ile (Zhang et al., 2017; Howe et al., 2018). We found similar splicing variants of the JAZ protein-coding genes in cotton. The *Gh_D01G1406*, *Gh_D05G1155*, and *Gh_D05G2675* contained the complete jas domains, while the RI splicing variants of these three genes only had partial jas motif (**Figure 7**). This indicated that such AS form of the JAZ genes was conserved in cotton and Arabidopsis. Interestingly, these GhJAZ RI-transcription were significantly increased by the two insect infestations and the change was more significant in HA samples (**Figure 7**). This suggested that the plant further coordinated the defense response to insects by regulating JAZ sensitivity to JA-Ile through AS.

## Discussion

In this study, we surveyed the plant defense response to the two insects of the different guilds. Leaf-chewing insects cause serious wounding damage on plants, quickly triggering JA-mediated signaling (Howe et al., 2018; Erb and Reymond, 2019). This is consistent with our observation that JA signaling is dominantly involved in cotton defense against *H. armigera*. The feeding process of *A. lucorum* is more complicated including mechanical and punctured damages, and delivering a large amount of insect oral secretions in the plant tissues. *A. lucorum* not only induces genes of the JA signal pathway but also genes related to plant disease resistance pathways which can be usually induced by SA (Pieterse et al., 2012). The predominant induction of some PR genes in AL samples might be at least partially responsible for the symptoms of leaf wilting and necrotic plaques in the *A. lucorum* infested cotton. Hormone signaling usually shaped the global gene expression profiling and the differential contributions of JA and SA in response to the two insects might responsible for the highly specialized response.

The SPIs genes ubiquitously present in plants and have various functional roles (Clemente et al., 2019). It has been well acknowledged that some plant SPIs are involved in insect defense(van der Hoorn and Jones, 2004; Santamaria et al., 2012), however, no PI protein with insecticide has been identified from cotton. Here, we found that there was a huge difference in the induction of protease inhibitors between the two insects. The *Gh_Sca005135G01* which strongly induced by *H. armigera* could significantly inhibit the growth of *H. armigera*, while *A. lucorum* induced SPI, *Gh_A11G1177*, could not. This indicated that the plant might defend different insects by inducing specialized protease inhibitors.

AS is a conserved gene regulation in eukaryotes and has been thought to be involved in many biological processes. In recent years the AS regulation in stress has been reported (Laloum et al., 2018; de Francisco Amorim et al., 2018; Calixto et al., 2018; Shih et al., 2019). Here, we found that most DAS genes caused by insect damage were not affected at the transcription level. Previous studies of defense usually focused on genes with significant changes in expression levels. Our study showed that a set of genes in plants respond to insect infestation by differentially AS suggesting the AS regulation is also required in defense.

Some JAZ protein-coding genes have conserved splicing patterns in Arabidopsis and cotton. The protein products of the variant AtJAZ RI-transcripts lacked the jas-domain and no longer respond to JA-Ile mediated degradation (Chung et al., 2010; Howe et al., 2018). The induction of GhJAZ RI-transcripts by *H. armigera* infestation in cotton might be important to avoid the overreaction to the JA signaling and minimize the negative impacts. The DAS events in HA and AL are largely different and the *H. armigera* has more impacts on AS in cotton than the *A. lucorum*. To date, it is well characterized that transcription is dominant in JA-mediated defense response to *H. armigera* (**Figure 2**). In the future, whether AS acts as a critical step to regulate JA signaling needs to be elucidated.

## Data Availability Statement

The datasets presented in this study can be found in online repositories. The names of the repository/repositories and accession number(s) can be found in the article/**Supplementary Material**.

## Author Contributions

Y-BM designed and supervised the research and wrote the article. D-YC and Q-YC performed most of the experiments and sequence analysis and assisted in writing. D-DW, Y-PM, and M-YW performed part of the experiments. J-RH assisted in data analysis and writing. D-YC and Q-YC contributed equally to this work.

## Funding

This research was supported by the Ministry of Science and Technology (2016YFA0500800); National Natural Sciences of China Grants 31772177 and 31788103; The Ministry of Agriculture of China Grant 2016ZX08009001-009; Chinese Academy of Sciences Grant QYZDY-SSW-SMC026; and the Strategic Priority Research Program of the Chinese Academy of Sciences Grant XDB11030000.

## Conflict of Interest

The authors declare that the research was conducted in the absence of any commercial or financial relationships that could be construed as a potential conflict of interest.
