# Supplementary material for: Differential Transcription and Alternative Splicing in Cotton Underly Specialized Defense Responses Against Pests
Source: Front Plant Sci. 2020 Sep 15;11:573131. doi: 10.3389/fpls.2020.573131 (PMC7533563; doi:10.3389/fpls.2020.573131)
Supplement: Supplementary file 1 [file DataSheet_1.docx]

**Supporting Information**

Article title: **Differential transcription and alternative splicing in cotton underly specialized defense responses against pests**

Authors: **Dian-Yang Chen; Qiu-Yi Chen; Dan-Dan Wang; Yu-Pei Mu; Mu-Yang Wang; Ji-Rong Huang; Ying-Bo Mao**

**The following Supporting Information is available for this article:**

**FIGURES**

**Figure S1:** Images of the untreated (A) and *A.lucorum* infested cotton plants (B-D).

Figure S2: Transcriptomes analysis of the untreated (CK), *H. armigera* (HA) and *A. lucorum* (AL) infested cotton cotyledons.

**Figure S3:** The expression of the PR genes with higher induction by *A.lucorum* from the RNA-Seq results*.*

Figure S4: The effects of protein inhibitor on cotton bollworm growth.

Figgure S5: The amount and distribution of the different types of alternative splicing events in cotton.

**TABLES**

**Table S1:** Primers used in this investigation.

**Table S2:** The rules for well-expressed AS genes selection.

**Table S3:** General information of the differentially expressed genes (DEGs) by insect infestations.

**Table S4:** Gene Ontology (GO) enrichment of insect down-regulated genes.

**Table S5:** Gene Ontology (GO) enrichment of the up-regulated DEGs from HA and AL.

**Table S6:** The DEGs with higher inductions by *H. armigera* (HA) and by *A.lucorum* (AL) were subject to GO enrichment assay respectively.

**Table S7:** The detailed information of the JA and SA related genes in Figure 2B.

**Table S8:** The detailed information of the PR genes in Figure 3.

**Table S9:** General information of the differential AS (DAS) events caused by the *H. armigera* and *A. lucorum* infestations in cotton.

**Table S10:** Gene Ontology (GO) enrichment of differential AS genes from HA samples and AL samples.

**Table S11:** PSI (Percent spliced in) value of U1 and SR proteins from CK samples, HA samples and AL samples.

**Table S 3 to 11:** See separate Excel file.


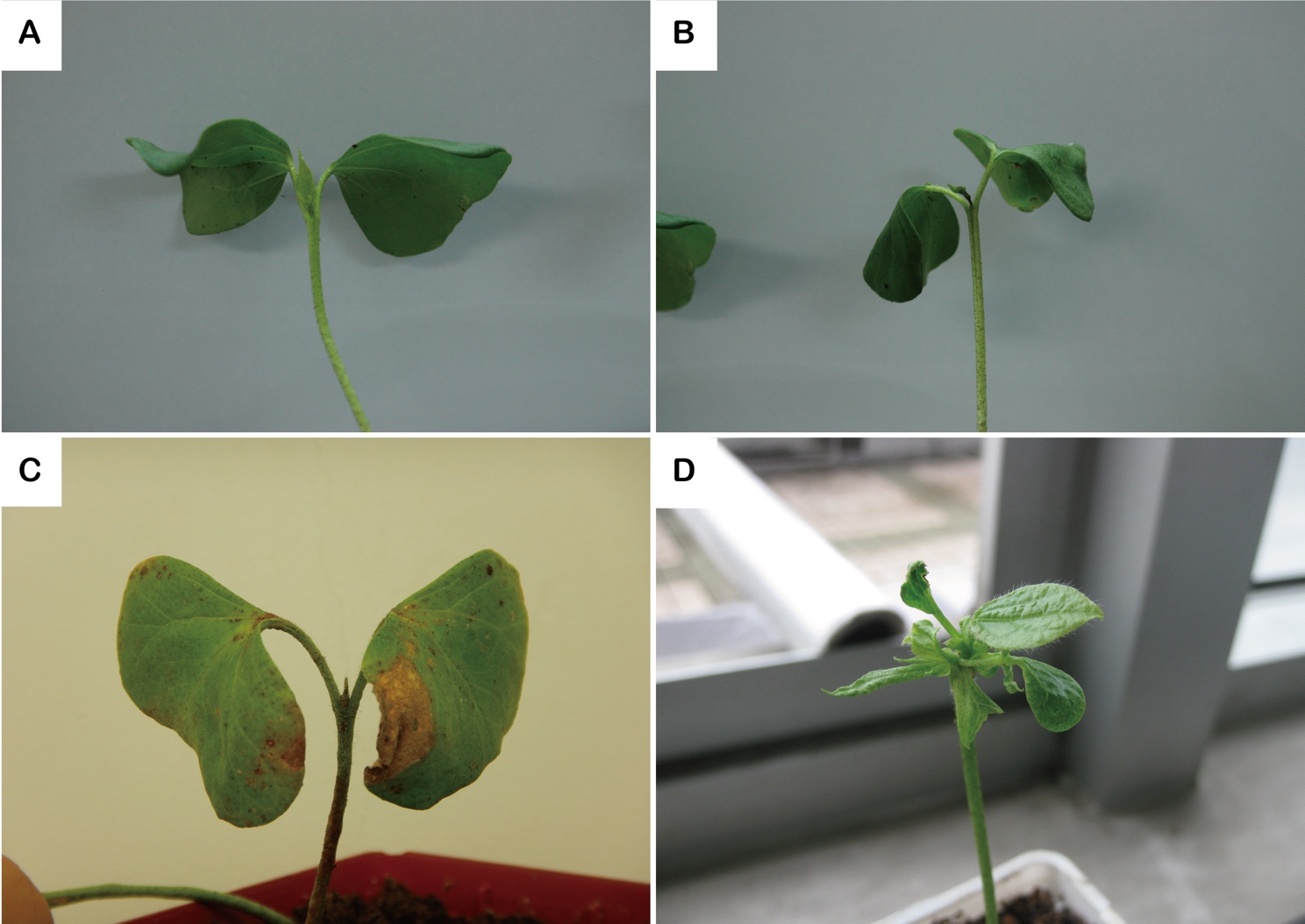


**Figure S1.** Images of the untreated (A) and *A.lucorum* infested cotton plants (B-D). The *A.lucorum* infested cotton seedlings exhibited symptoms of leaf wilting (B), necrotic plaques (C) and abnormal leaf development (D)


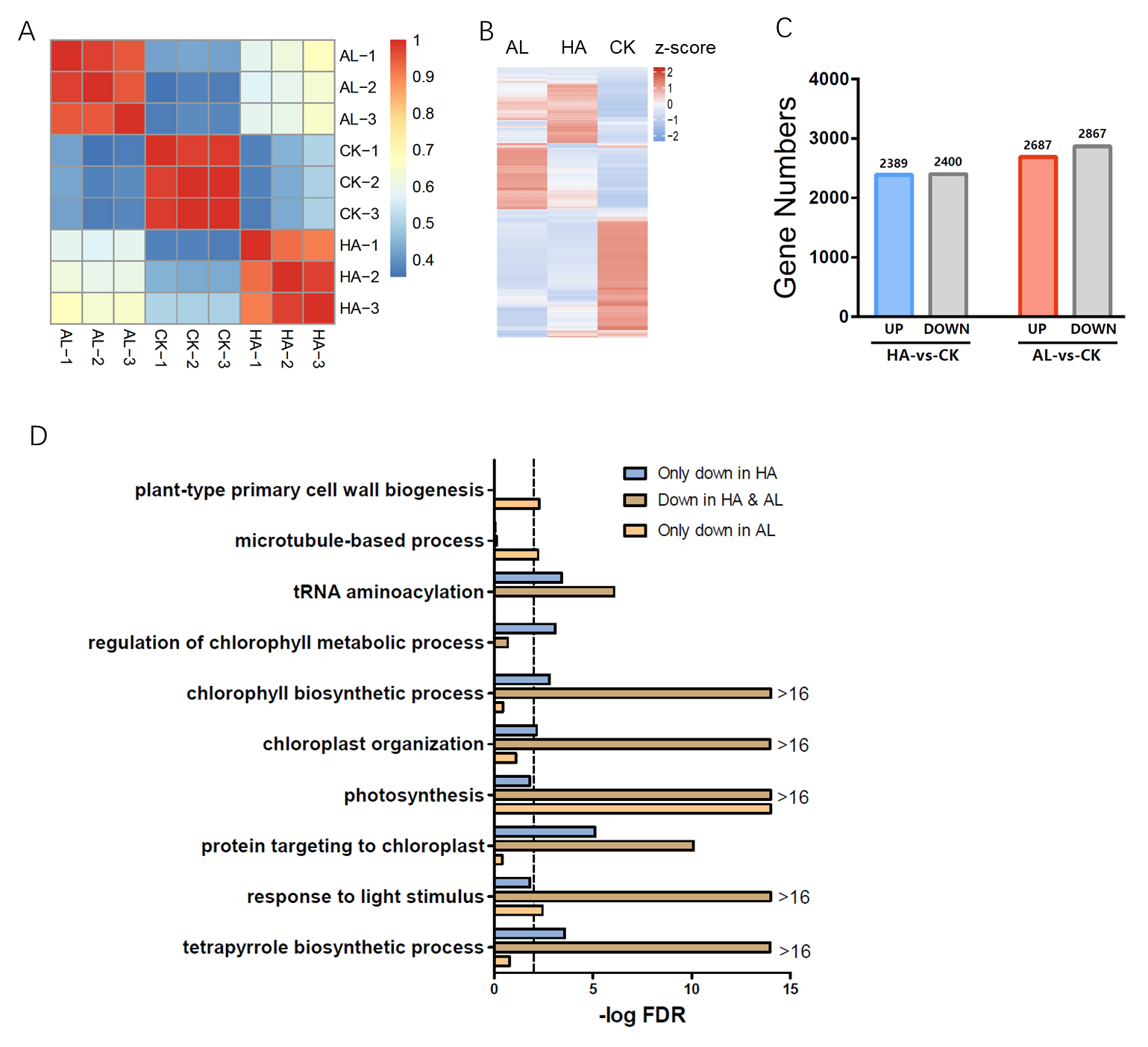


Figure S2. Transcriptomes analysis of the untreated (CK), *H. armigera* (HA) and *A. lucorum* (AL) infested cotton cotyledons. A. Corelationship analyze of transcriptome row data of CK, HA and AL sample groups. It suggested that *H. armigera* and *A. lucorum* contributed to differential transcription of cotton. B. Heat map analysis of the bulk RNA-seq data. The 3 biological replicates (-1,-2,-3) of CK, HA and AL sample groups were analyzed. High uniformity within the 3 biological replicates of the same sample group and quite different among the CK, HA and AL were observed. C. The amount of up and down-regulated genes by the *H. armigera* and *A. lucorum* infestations. D. The GO enrichment assay of insect down-regulated genes. The down-regulated genes only in HA, in AL and in both HA & AL were separately subject to GO enrichment assay.


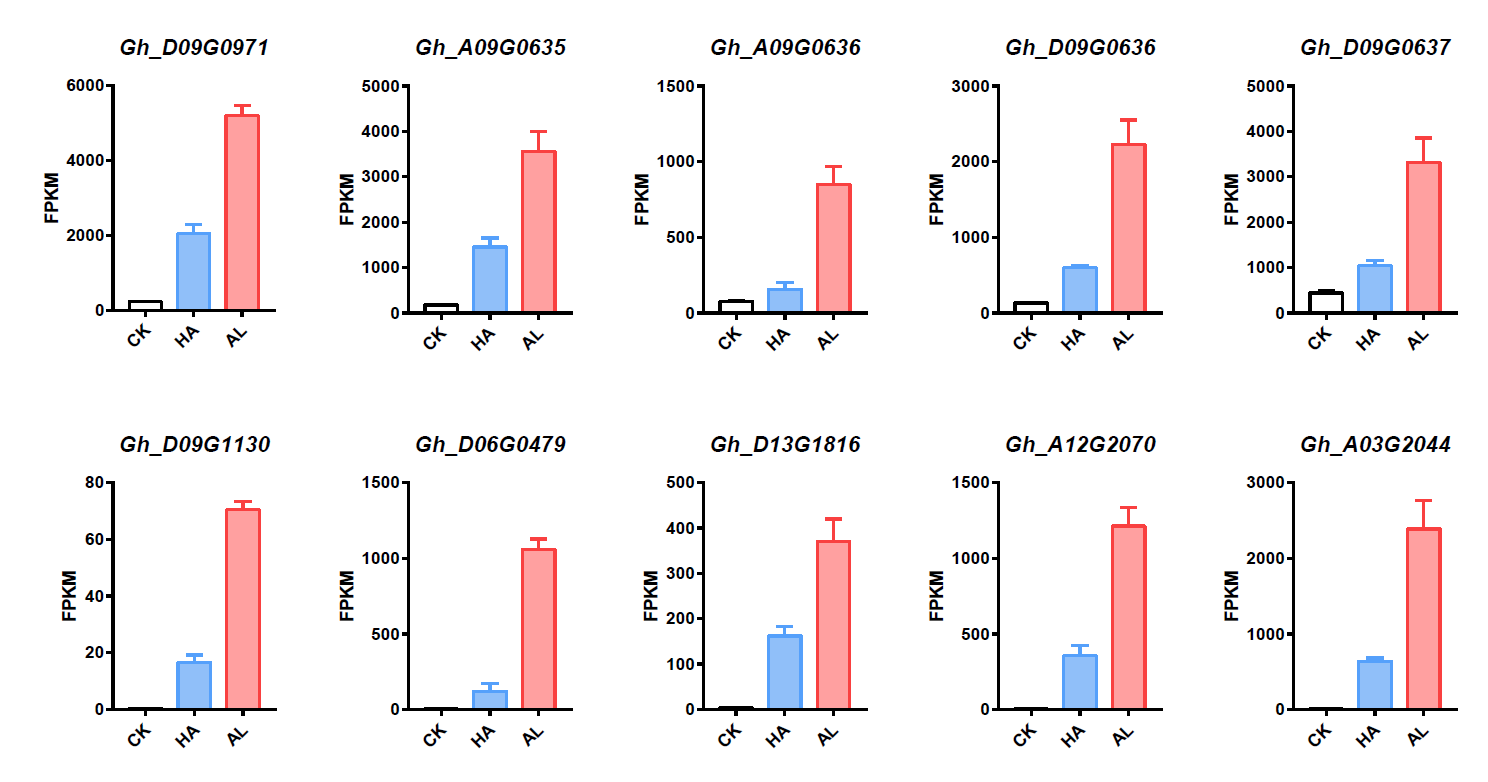


Figure S3. The expression of the PR genes with higher induction by *A.lucorum* from the RNA-Seq results*.* HA and AL indicated cotton seedlings treated by *H. armigera* and *A. lucorum* respectively. Error bar means ± SEM (n=3 biological replicates).


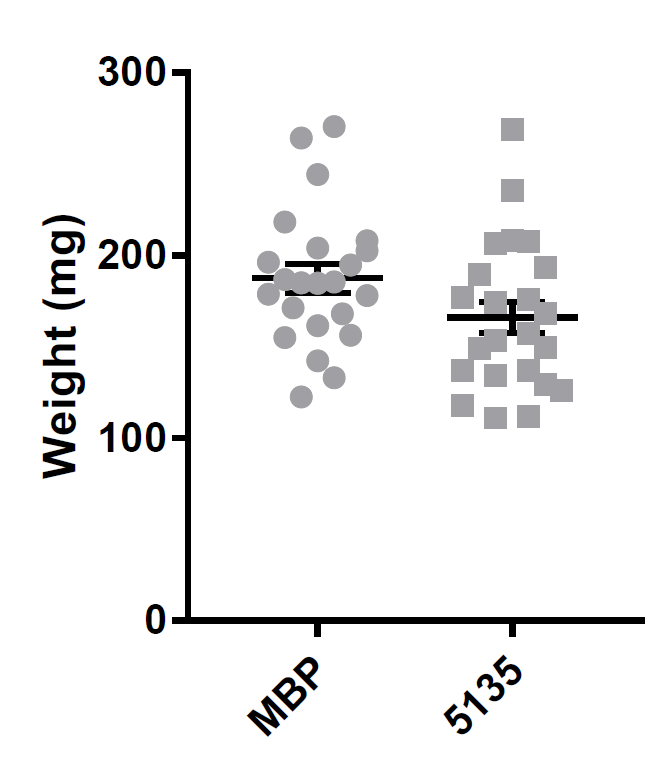


Figure S4. The effects of protein inhibitor (Gh_Sca005135G01) on cotton bollworm growth. The 2nd instar larvae were fed with basal artificial diet mixed with MBP and Gh_Sca005135G01 expressing E. coli cells for4days and the weight of each individual was detected. Error bar means ± SEM (n=30-35).


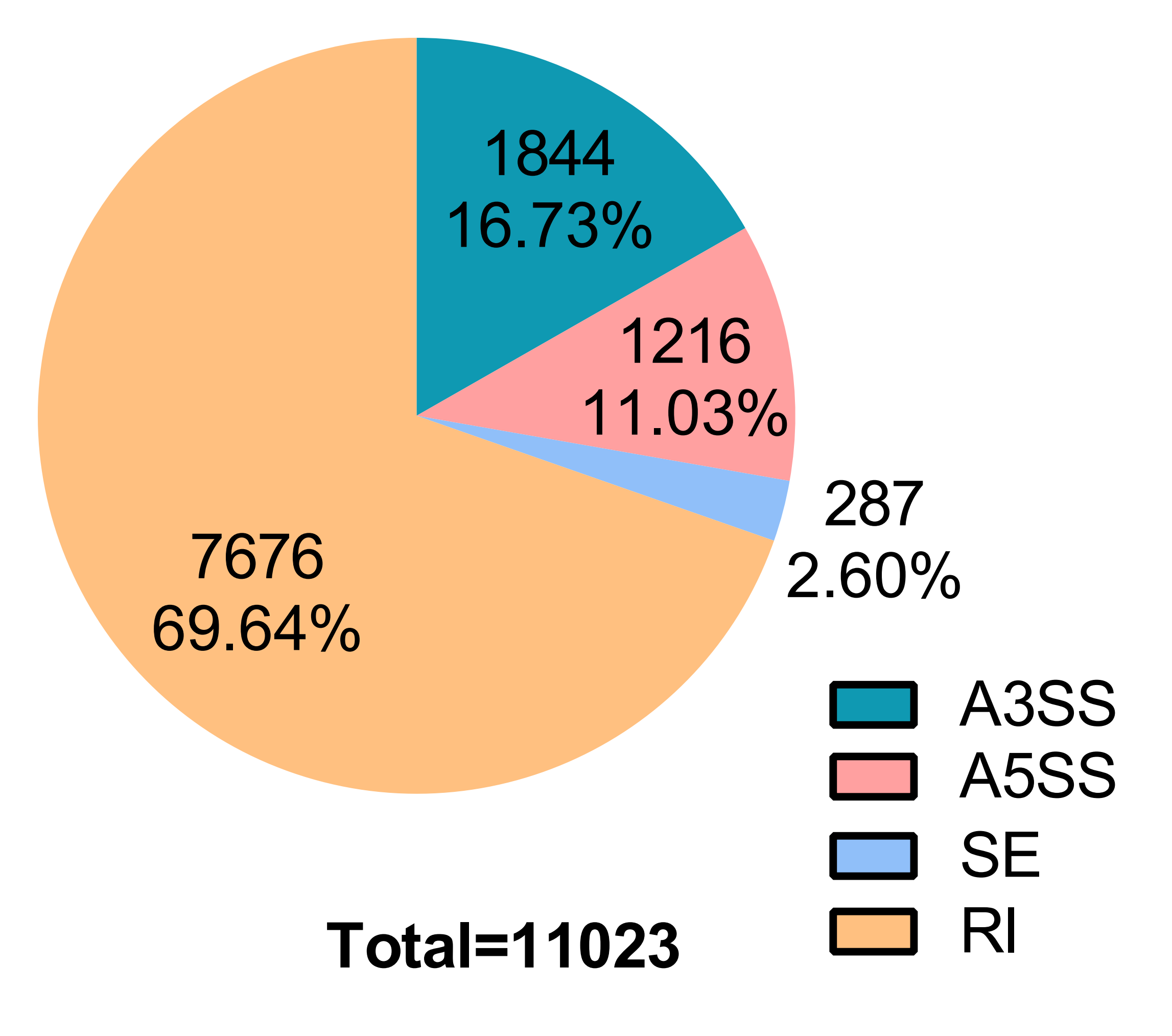


Figure S5. The amount and distribution of the different types of alternative splicing events in cotton. A3SS, alternative 3' splice site; A5SS, alternative 5' splice site; SE, skipping exon; RI, retained intron.

**Table S1** Primers used in this investigation.

| primer for qPCR | | |
| --- | --- | --- |
|  | primer F (5'-3') | primer R (5'-3') |
| GhHIS3 | CGGTGGTGTGAAGAAGCCCTAT | AATTTCACGAACAAGCCTCTGGAA |
| Gh_D01G1683 | TAGCCCAAACTTCCCATGAA | CCCGTAGTTGTAGTTCCAA |
| Gh_A05G1019 | AACACAACCATCTTCCTCCTTCTC | TTTGCCGAAGTCTGGAGTC |
| GH_A10G2244 | GCGGACAAGGTGAAGG | CGTCGGGTTTAGGTGG |
| GH_A06G0705 | CTCCTCCTCGGAAACT | TCCACTGAAGAACCCA |
| Gh_D12G2247 | AAATCTCCTCCCTCCT | GTCCACCACAATCACC |
| Gh_D10G2219 | ACATTGATGGCAACGAGG | AGTTTGGGTCTGAGGGGA |
| Gh_D09G1520 | AAGTGCTTTCCTACCC | AGATTTTGCCCCATAA |
| Gh_D06G1912 | CGACTTCTGCTTGTTATC | TGTCTTGAATGGGTGTTA |
| Gh_D03G1462 | TTGACATCTCCAACATC | ACCTCTCCTTGAAAAAC |
| Gh_D10G1757 | AGCACCAGATAGGAGG | TAGCCAAGAGGTAAGC |
| Gh_A13G2341 | AACCTGCCAGCACATTA | AGTCCTTGAAAACCCGA |
| Gh_A10G2355 | CGTTGTTCGCCTATCC | TCACCCACCATTTTCC |
| Gh_D09G0971 | TGACTTTTCAGGCACT | CACCTCACCTTAGCAC |
| Sca005135G01 | CCATCGTCCTTTTCGT | ACCGTTGTTGTTTCGC |
| primer for protein expression in E.coli cells | | |
|  | primer F (5'-3') | primer R (5'-3') |
| 5135-Pmal-c5x | GGGATCGAGGGAAGGATGAAAACCACAACAG | GGACATATGTGAAATCTAATTATGAACAACTTGT |
| 1177-Pmal-c5x | GGGATCGAGGGAAGGATGACAGATTGCATCG | GGACATATGTGAAATCTAGCCAATGTGAGGCGTG |

**Table S2** The rules for well-expressed AS genes selection.

| **IJC-CK** | **SJC-CK** | **IJC-HA** | **SJC-HA** | **IJC-AL** | **SJC-AL** |
| --- | --- | --- | --- | --- | --- |
| Χ | Χ | Χ | Χ | Χ | Χ |
| Χ | Χ | Χ | Χ | Χ | Ο |
| Χ | Χ | Χ | Χ | Ο | Χ |
| Χ | Χ | Χ | Ο | Χ | Χ |
| Χ | Χ | Ο | Χ | Χ | Χ |
| Χ | Χ | Ο | Χ | Ο | Χ |
| Χ | Χ | Χ | Ο | Χ | Ο |
| Χ | Ο | Χ | Χ | Χ | Χ |
| Χ | Ο | Χ | Χ | Χ | Ο |
| Χ | Ο | Χ | Ο | Χ | Χ |
| Χ | Ο | Χ | Ο | Χ | Ο |
| Ο | Χ | Χ | Χ | Χ | Χ |
| Ο | Χ | Χ | Χ | Ο | Χ |
| Ο | Χ | Ο | Χ | Χ | Χ |
| Ο | Χ | Ο | Χ | Ο | Χ |

If the IJC values > 5 in all the three replicates, marked the IJC “Ο”, otherwise “Χ”. If the SJC values > 5 in all the three replicates, marked the SJC “Ο”, otherwise “Χ”. Remove the AS events with the combinations listed in the table below and the rested AS events were subject to further analysis.
